# Adult psychiatric inpatient admissions and length of stay before and during the COVID-19 pandemic in a large urban hospital setting in Vancouver, British Columbia

**DOI:** 10.3389/frhs.2024.1365785

**Published:** 2024-05-14

**Authors:** Angela Russolillo, Michelle Carter, Mejiao Guan, Pulkit Singh, David Kealy, Julia Raudzus

**Affiliations:** ^1^ Department of Psychiatry, Providence Health Care, Vancouver, BC, Canada; ^2^ Faculty of Health Sciences, Simon Fraser University, Burnaby, BC, Canada; ^3^ School of Nursing, University of British Columbia, Vancouver, BC, Canada; ^4^ Statistics and Health Economics, Centre for Advancing Health Outcomes, Vancouver, BC, Canada; ^5^ Department of Psychiatry, University of British Columbia, Vancouver, BC, Canada

**Keywords:** COVID-19, hospitalizations, health services, mental health, substance use, length of stay

## Abstract

**Introduction:**

During the COVID-19 pandemic individuals with mental illnesses faced challenges accessing psychiatric care. Our study aimed to describe patient characteristics and compare admissions and length of stay (LOS) for psychiatric-related hospitalizations before and during the COVID-19 pandemic.

**Methods:**

We conducted a retrospective analysis using health administrative data comparing individuals with an acute psychiatric admission between two time periods: 1st March 2019 to 31st December 2019 (pre-COVID) and 1st March 2020 to 31st December 2020 (during-COVID). Multivariable negative binomial regression was used to model the association between most responsible diagnosis type and the two-time periods to hospital LOS, reporting the Rate Ratio (RR) as the measure of effect.

**Results:**

The cohort comprised 939 individuals who were predominately male (60.3%) with a severe mental illness (schizophrenia or mood-affective disorder) (72.7%) and a median age of 38 (IQR: 28.0, 52.0) years. In the multivariable analysis, anxiety disorders (RR: 0.63, CI: 0.4, 0.99) and personality disorders (RR: 0.52, CI: 0.32, 0.85) were significantly associated with a shorter LOS when compared to individuals without those disorders. Additionally, when compared to hospital admissions for non-substance related disorders the LOS for patients with substance-related disorders were significantly shorter during the COVID period (RR: 0.45, CI: 0.30, 0.67) and pre-COVID period (RR: 0.31, CI: 0.21, 0.46).

**Conclusions:**

We observed a significant difference in the type and length of admissions for various psychiatric disorders during the COVID-19 period. These findings can support systems of care in adapting to utilization changes during pandemics or other global health events.

## Introduction

The COVID-19 pandemic placed unprecedented stress on both individuals and healthcare systems worldwide. Social isolation, financial stress and fear contributed to exacerbation of existing psychiatric conditions (1) and increased incidence of mental disorders (2, 3). Vulnerable groups, including individuals with pre-existing mental disorders, were disproportionally impacted by societal restrictions and mandated public health measures (4). Faced with escalating COVID-19 transmission rates, many jurisdictions required patient and health care provider isolation, resulting in a shift towards virtual healthcare and telemedicine to promote continuity of care while maintaining the safety of patients and providers (5–7). Despite rapid implementation of virtual services, reports of decreased availability and potential inadequacies of mental health treatment during the pandemic were a major concern and may have impacted access to those seeking psychiatric care (8).

There is emerging international evidence of variation in healthcare utilization patterns for individuals with mental illness during the COVID-19 pandemic. Italy and Portugal reported a decrease in psychiatric hospitalizations (9, 10), yet indicators of psychological distress such as suicidal ideation were notably increased in some jurisdictions (11). Similar trends of reduced utilization were observed across emergency department presentations in Germany (12) and Switzerland (13), but post-lockdown periods were associated with increased presentations and more severe clinical conditions (14). Moreover, observational research demonstrates varying hospitalization trends across diagnostic groups and population subgroups. In the United States (US) population-level research identified a significant increase in the number of admissions and longer length of inpatient hospitalizations for individuals with eating disorders during the pandemic (15). In addition, US youth and pediatric populations were linked to increased severity of symptoms (16) and higher rates of hospital admissions during the pandemic when compared to pre-pandemic periods (17).

Across Canada, mental health and substance use service utilization during the pandemic has mostly suggested a decline in emergency department and hospital visits (18, 19) with hospitalization rates returning to pre-pandemic levels by March 2021 (20). Evidence from Ontario, Canada using population-level administrative data found psychiatric-related hospital admissions did not return to pre-restriction rates (21) and while overall volumes may have decreased, monthly increases were observed for specific mental disorders post pandemic (22, 23). In British Columbia (BC), Canada COVID-19 mental-health related service use rose sharply during 2020, but quickly stabilized; however, other types of mental health service use and drug dispensation continued to rise significantly into 2021 (24). The impact of the pandemic on mental health hospital admissions within Canada exhibited considerable variability across geography and disorder type and research examining changes to hospital LOS for psychiatric patients during COVID-19 in Canada remains scare. Despite what appears to be a stabilization of psychiatric hospitalization trends, it remains critical to evaluate the impact of changes to acute care (i.e., hospital-based care and treatment for a disease or severe episode of illness on a time limited basis) (25) and explore other health system indicators including length of stay (LOS) during the COIVD-19 period. Hospital LOS, among other indicators is commonly used to measure aspects of hospital quality and efficiency (26, 27) and is a standard metric in many health systems to understand operational needs including flow of patients through hospital services (28). While commonly used, hospital LOS is complex and can be affected by several factors including illness severity, treatment complexity and individual patient characteristics (29, 30) which makes calculating and predicting an optimal LOS challenging (31). Average LOS for psychiatric inpatients generally exceeds other medical specialities (32–34), with shorter LOS potentially signalling treatment disparities or perpetuating a “revolving door” phenomenon (35, 36). Nevertheless, it is generally accepted that reducing LOS for patients is desirable as longer than average LOS is also associated with increased risk of hospital acquired harms (e.g., falls, infection, other adverse events) and healthcare resources utilization (37, 38).

In BC, as in numerous other regions, the pandemic was associated with significant unmet health needs and worsening mental health and substance use outcomes (39). Regrettably, health system capacity remains strained since the pandemic (40, 41) and health services planning has failed to examine how to address changes in demand for health services following COVID-19 (42, 43). Moreover, such planning needs to be informed by service utilization data that are representative of local contexts and populations. To address this gap we conducted a retrospective cohort study using health administrative data comparing acute psychiatric admissions and LOS before and during the COVID-19 pandemic in Vancouver, BC, Canada.

## Methods

### Design, setting, participants and data source

In BC, Canada the local government declared a provincial state of emergency on 17 March 2020, to support the provincewide response to the COVID-19 pandemic. Schools, playgrounds and non-essential businesses were closed, and social distance requirements were broadly implemented (44). Concurrently, healthcare organizations limited visitation and implemented new safety protocols while also postponing or delaying nonessential care (45). Despite a brief disruption in healthcare services (40), by May 2020 local hospitals (including the study site) adapted services in response to provincial COVID-19 policies and implemented site specific screening, testing and infection control precautions. Additionally, a coordinated response was developed and implemented to manage COVID-19 infections among psychiatric patients who required hospital admission. Our study site created designated psychiatric units for suspected and confirmed COVID-19 patients to receive psychiatric care while minimizing the risk of transmission and disruptions to treatment.

This retrospective cohort study used health administrative data for adults with a psychiatric admission to a single urban hospital in Vancouver, BC. Admission rates and LOS were compared between two time periods: 1st March 2019 to 31st December 2019 (pre-COVID) and 1st March to 31st December 2020 (during-COVID). Our study site provides inpatient and emergency services to a catchment area that includes Vancouver's Downtown Eastside, a region with high rates of mental illness, substance use, and communicable disease (46). The patient's first discharge during that time period determined the index admission (first hospital admission during observation period). Hospital LOS (number of days a patient was admitted to hospital) was measured for each hospital admission during the study period. Patients were excluded from the study if they had an invalid personal health number, invalid age, were younger than 17 years of age, died during the psychiatric hospitalization or transferred to another hospital at the time of discharge. Sociodemographic variables (age, sex, and homelessness) were obtained from electronic medical records. Hospitalization data were obtained from the Ministry of Health's Discharge Abstract Database, which includes information related to each acute hospital separation. The study was reviewed and approved by the University of British Columbia—Providence Health Care (UBC-PHC) Research Ethics Board–H21-00462. Because of the retrospective nature of this study, patient informed consent for inclusion was waived by the institutional review board of UBC-PHC Research Ethics Board.

### Statistical analysis and outcomes

Our primary outcome was any psychiatric hospital admission and hospital LOS. LOS is a continuous variable indicating the number of days a patient was admitted to hospital (total number of days from admission date to discharge date). All hospitalization records included diagnostic codes representing the most responsible diagnosis for each psychiatric hospitalization. The present study used International Statistical Classification of Diseases and Related Health Problems, Tenth Revision, Canada (ICD-10-CA) to determine the most responsible diagnosis, a modified version of ICD-10, developed by Canadian Institute for Health Information and used across Canada. The following ICD-10-CA codes were used to determine the most responsible diagnoses for hospitalizations associated with: schizophrenia, delusional and non organic psychotic disorders F20 (excluding F20.4) to F25, F28, F29, F53.1; mood/affective disorders (F30–F34, F38, F39, F53.0); anxiety disorders (F40–F43, F48.8, F48.9); personality disorders (F60–F62, F69, F21); and substance use disorders (F55, F10–F19).

Patient demographic variables were summarized and compared using percentages and Chi-square tests for categorical variables (sex, homelessness) and median (IQR) and Kruskal–Wallis tests for continuous variables (age and LOS), stratified by the pre-COVID and during-COVID periods. Given the observed overdispersion, we selected the Negative Binomial model to evaluate the impact of diagnosis type on LOS. The negative binomial model was adjusted for age, sex, homelessness and pre-during COVID, where interactions between diagnoses type (yes vs. no) and the two time periods (pre-COVID vs. during COVID) on hospital LOS were tested. Significance for the interaction was determined with a *p*-value of <0.1. We used Rate Ratios and 95% CIs to estimate the association of predictor variables on the outcome. Analyses were completed used SAS, version 9.4.

## Results

**Figure 1:**
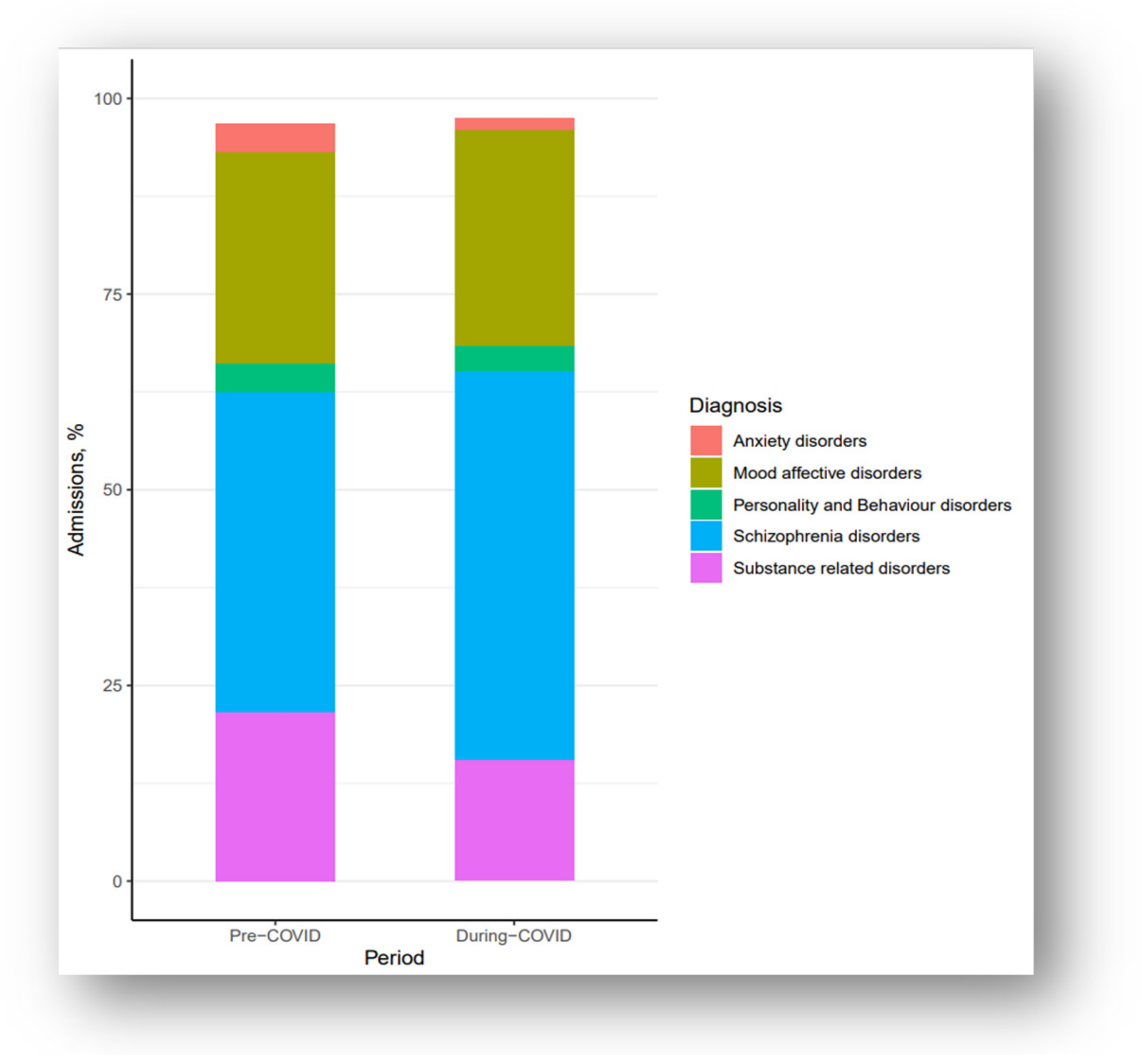
Proportion of hospital admissions by diagnosis type for the pre-COVID period (2019; *n* = 472) and during-COVID period (2020; *n* = 467) from an urban hospital in Vancouver, BC (*n* = 939).

The cohort comprised 939 patients who were predominately male (60.3%) with a severe mental illness (schizophrenia or mood-affective disorder) (72.7%) and a mean age of 38 (IQR: 28.0, 52.0) years. There were 472 acute psychiatric admissions during the pre-COVID period and 467 acute psychiatric admissions during the COVID period. After adjusting for the population size, the pre-COVID admission rate was 11.1 per 100,000 individuals and the during-COVID admission rate was 10.9 per 100,000 individuals. In the during-COVID period, admissions related to schizophrenia and psychotic disorders were significantly higher (49.7% vs. 40.9%; *p* = 0.007) and admissions related to substance disorders were significantly lower (15.4% vs. 21.6%; *p* = 0.015) when compared to the pre-COVID period (Figure 1). Additional details characterizing the sample during the pre and during COVID periods are reported in Table 1.

**Table 1:** Characteristics of included patients at the pre-COVID period (2019) and during-COVID period (2020), from an urban hospital in Vancouver, BC (*n* = 939).

Variables	All patients (*N* = 939)	Pre-COVID (*N* = 472)	During-COVID (*N* = 467)	*P*-value	Missing
Age, median (IQR)	38.0 (28.0, 52.0)	38.0 (29.0, 53.0)	38.0 (28.0, 51.0)	0.503	0
Sex, *n* (%)				0.237	3
Female	371 (39.6)	176 (37.4)	195 (41.8)		
Male	565 (60.3)	294 (62.4)	271 (58.2)		
LOS, median (IQR)	12.0 (5.0, 24.0)	12.5 (5.0, 27.0)	12.0 (5.0, 22.0)	0.307	
Schizophrenia, delusional and non-organic psychotic disorders, *n* (%)	425 (45.3)	193 (40.9)	232 (49.7)	0.007	0
Substance-related disorders, *n* (%)	174 (18.5)	102 (21.6)	72 (15.4)	0.015	0
Mood-affective disorders, *n* (%)	257 (27.4)	128 (27.1)	129 (27.6)	0.862	0
Anxiety disorders, *n* (%)	24 (2.6)	17 (3.6)	7 (1.5)	0.041	0
Adult personality and behaviour disorders, *n* (%)	32 (3.4)	17 (3.6)	15 (3.2)	0.742	0
Homelessness, *n* (%)	136 (14.5)	71 (15.0)	65 (13.9)	0.625	0

The median pre-COVID and during-COVID hospital LOS was 12 days. As shown in Table 2, female patients had a longer LOS than male patients (*p* = 0.022) and LOS increased with age (*p* ≤ 0.001). When compared to individuals without each disorder type, individuals with schizophrenia-related disorders and mood-affective disorders had longer lengths of stay and individuals with substance-related disorders, anxiety disorders, or personality and behaviour disorders had shorter lengths of stay (Table 2). Comparisons of hospital LOS for each disorder are provided in (Supplementary Material Figures S1–S5).

**Table 2:** Distribution of hospital LOS^a^ for the pre-COVID period (2019) and during-COVID period (2020), from an urban hospital in Vancouver, BC (*n* = 939).

Variable	Hospital LOS (days)								*P*-value
	N	Min	Q1	Mean	SD	Median	Q3	Max	
Pre/during COVID									0.307
Pre-COVID (March 2019–December 2019)	472	1	5	20.6	24.5	12.5	27	190	
During-COVID (March 2020–December 2020)	467	1	5	16.6	16.7	12	22	153	
Age									<0.001
≤20	33	1	3	17.8	32.9	10	21	33	
(20, 30)	258	1	4	15.5	18.7	10	18	258	
(30, 40)	217	1	5	17.0	18.5	11	23	217	
(40, 50)	175	1	5	17.5	19.5	13	22	175	
(50, 60)	140	1	5	20.3	22.0	14.5	27	140	
(60, 70)	81	1	8	23.7	20.4	20	34	81	
>70	35	9	19	39.2	30.3	30	50	35	
Sex									0.022
Female	371	1	6	20.0	22.1	14	26	371	
Male	565	1	4	17.7	20.4	11	24	565	
Schizophrenia, delusional and non-organic psychotic disorders									<0.001
No	514	1	4	16.0	19.9	10	21	190	
Yes	425	1	7	21.7	22.0	14	30	171	
Substance-related disorders									<0.001
No	765	1	7	21.0	22.2	15	27	190	
Yes	174	1	3	7.8	9.5	4	9	62	
Mood-affective disorders									<0.001
No	682	1	4	17.7	20.9	11	24	171	
Yes	257	1	8	21.0	21.5	16	25	190	
Anxiety disorders									0.051
No	915	1	5	18.8	21.2	12	24	190	
Yes	24	1	3	12.2	13.0	6.5	17.5	48	
Adult personality and behaviour disorders									0.021
No	907	1	5	18.8	21.1	12	24	190	
Yes	32	1	2	13.9	21.3	8	16	117	
Homelessness									0.089
No	803	1	5	19.2	22.0	13	25	190	
Yes	136	1	5	14.9	14.4	11	19	77	

Min, minimum; SD, standard deviation; Max, maximum.

^a^ LOS for each diagnosis type combines both the pre-COVID and during-COVID periods.

In unadjusted analysis the mean LOS decreased by 20% during-COVID when compared to the pre-COVID period (RR: 0.80, CI: 0.71, 0.91) (Table 3). Except for adult personality and behaviour disorders, all the other diagnosis types are significantly associated to LOS (*p* ≤ 0.05). Results for the multivariable negative binomial model are shown in Table 4. Anxiety disorders (RR: 0.63, CI: 0.40, 0.99) and adult personality (RR: 0.52, CI: 0.32, 0.85) disorders were significantly associated with a shorter LOS when compared to individuals without those disorders at the pre- and during-COVID periods. Additionally, having a substance use disorder was significantly associated with a shorter LOS. There was a significant interaction between substance-related disorders and pre-during COVID period (*p*-value = 0.057) (See Supplementary Material Table S1). The ratio of LOS among patients with and without substance-related disorders increased from RR: 0.31, CI: 0.21, 0.46 in the pre-COVID period to RR: 0.45, CI: 0.30, 0.67 in the during COVID period (Table 4). There were no significant associations between LOS and schizophrenia-related disorders or mood and affective disorders.

**Table 3:** Univariate associations between diagnosis type and hospital LOS (days) from an urban hospital in Vancouver, BC, 2019–2020.

Variable	Estimate (95% CI)	RR (95% CI)	*P*-value
Age, per 1-year increases	0.01 (0.01, 0.02)	1.01 (1.01, 1.02)	<.0001
Male	−0.13 (−0.25, 0)	0.88 (0.78, 1)	0.0491
During-COVID	−0.22 (−0.34, −0.1)	0.80 (0.71, 0.91)	0.0004
Homelessness	−0.25 (−0.43, −0.08)	0.78 (0.65, 0.92)	0.0046
Schizophrenia, delusional and non-organic psychotic disorders	0.30 (0.18, 0.42)	1.35 (1.2, 1.53)	<.0001
Substance-related disorders	−0.99 (−1.14, −0.83)	0.37 (0.32, 0.44)	<.0001
Mood-affective disorders	0.17 (0.04, 0.31)	1.19 (1.04, 1.36)	0.0135
Anxiety disorders	−0.43 (−0.83, −0.04)	0.65 (0.44, 0.96)	0.0322
Adult personality and behaviour disorders	−0.30 (−0.64, 0.04)	0.74 (0.53, 1.04)	0.0821

RR, rate ratio; CI, confidence interval.

**Table 4:** Multivariable negative binomial model estimating the association between diagnosis type and hospital LOS^a^ (days) from an urban hospital in Vancouver, BC, 2019–2020.

Variable	Estimate (95% CI)	RR (95% CI)	*P*-value
Age, per 1-year increases	0.01 (0.01, 0.01)	1.01 (1.01, 1.01)	<.0001
Male	−0.09 (−0.21, 0.03)	0.91 (0.81, 1.03)	0.1440
Homelessness	−0.14 (−0.3, 0.03)	0.87 (0.74, 1.03)	0.1048
Schizophrenia, delusional and non-organic psychotic disorders	0.01 (−0.34, 0.35)	1.01 (0.71, 1.42)	0.9692
Mood-affective disorders	−0.10 (−0.45, 0.25)	0.90 (0.64, 1.28)	0.5728
Anxiety disorders	−0.65 (−1.14, −0.16)	0.63 (0.40, 0.99)	0.0095
Adult personality and behaviour disorders	−0.47 (−0.92, −0.01)	0.52 (0.32, 0.85)	0.0454
Substance-related disorders pre-COVID	−1.16 (−1.54, −0.78)	0.31 (0.21, 0.46)	<.0001
Substance-related disorders during-COVID	−0.81 (−1.20, −0.40)	0.45 (0.30, 0.67)	<.0001

RR, rate ratio; CI, confidence interval.

^a^ Adjusted for age, sex, and pre-during COVID and homelessness.

## Discussion

We observed statistically significant changes in hospital admissions and hospital LOS for several mental and substance use disorders during the COVID-19 period. When compared to pre-pandemic psychiatric hospital admissions by diagnoses, admissions for schizophrenia and psychotic disorders were significantly increased while admissions for substance disorders were significantly decreased. With respect to hospital LOS, anxiety disorders, personality disorder and substance use disorder were associated with significantly shorter LOS when compared to individuals without those disorders. To our knowledge, this is the first study to examine changes to acute psychiatric admissions and hospital LOS during the COVID-19 pandemic using clinical and administrative data from a large urban hospital in Vancouver, BC.

The pandemic had a disproportionate impact on individuals with severe mental illness and related vulnerabilities (e.g., homelessness, substance use), exacerbating symptoms and worsening clinical outcomes (1). Despite this trend, our study revealed an overall reduction in acute psychiatric hospitalizations during the pandemic when compared to the same time period in 2019. Possible explanations for this decrease in hospitalizations may be linked to public health restrictions limiting movement and deterring people from seeking help for non-COVID related illnesses during the pandemic (47). While our overall hospitalization rates declined, we observed a 10% increase in admissions related to psychotic disorders during the COVID-19 period. This rise in admissions may be attributed to the limited availability of community supports and the potentially heightened severity of individual needs requiring urgent or involuntary admissions (12) and psychotropic medications (24). Conversely, when compared to the pre-COVID period, we observed a decrease in substance-related admissions. While several factors such as restricted access to substances, disruptions in treatment services, or changes in substance use patterns during the pandemic (48, 49) provide context to the change in hospital admission rates in our study, mortality from drug-related deaths rose sharply during this period in BC (50). In 2020, the BC Coroners Service reported a staggering 80% rise in drug-related deaths in comparison to the preceding year, with a notable increase observed within the Vancouver Coastal Health (VCH) region—an area encompassing our study site (51). Fentanyl, a powerful synthetic opioid, was present in 84% of unregulated drug deaths in the VCH region in 2020, a slight increase from 81% in 2019 (51). The rise in fentanyl or fentanyl analogues involved deaths is growing and is currently the leading cause of opioid related deaths in BC and Canada (52). Continued assessment is crucial to understand the effects on individuals who may have received inadequate support during the pandemic, considering the ongoing challenges posed by the opioid crisis in British Columbia.

Risk factors for hospital LOS serve as important indicators for health system planning, resources allocation and costs. Healthcare delivery has undergone significant shifts and challenges in the wake of the COVID-19 pandemic, with the pandemic introducing unique variables and considerations that may influence LOS patterns. For example, our study site's adjustments to hospital policies regarding COVID-19 screening and quarantine for psychiatric patients reduced the need to transfer or prematurely discontinue care. This was achieved by setting up specialized psychiatric units specifically for patients who were suspected of having, or who tested positive for, COVID-19. Our findings highlight a 20% decrease in mean LOS during-COVID when compared to the pre-COVID period and demonstrate significant associations between important demographic factors and increased LOS. Consistent with prior research reporting gender disparities in LOS (53), we found that female patients had longer LOS compared to male patients, with a mean difference of 2.3 days. While propensity for help-seeking (54) and pre-existing rates of depression and anxiety are heightened among females (55, 56), rates of psychological distress during the pandemic were significantly higher for females (57) when compared to males. These sex differences may offer potential explanation to the increased hospital LOS for females in our study. Additionally, our results demonstrated that increasing age was associated with a longer LOS, with a small but statistically significant increase. This finding aligns with existing research reporting a robust association between older age and increased LOS in both general and psychiatric populations (29, 58). Extensive research on hospital LOS in psychiatric inpatients reveals a body of evidence indicating that longer stays are consistently linked to more adverse outcomes (e.g., suicidality and reduced social functioning, employment and housing) and escalating costs (59–61). Conversely, shorter than average hospital LOS might be indicative of lower quality care and associated with risk for early readmission (36, 62); however, this conclusion is not supported by current evidence (63). While determining an optimal LOS is complex, stabilization and coordinated discharge remains the goal of inpatient psychiatric care. Our study contributes to the growing body of evidence examining risk factors for LOS and provide insights into healthcare resource management while underscoring the importance of targeted interventions to address demographic disparities and promote equitable patient care.

In addition to socio-demographic factors, we found a disproportionate impact on hospital LOS for certain diagnostic subgroups. Specifically, individuals with anxiety disorders and adult personality disorders had significantly shorter hospital LOS compared to those without these disorders during the COVID-19 period. These findings align with prior evidence reporting shorter hospital stays for patients due to infection control and changes to treatment approaches, including the increased use of tele-psychiatry (64, 65). Despite reductions in acute care capacity, outpatient mental health services rapidly adapted to support patients using virtual health solutions and were associated with improved visit adherence over time and have emerged as a promising model for improving the efficiency of mental health care delivery (66, 67). Moreover, substance use disorders were significantly associated with a shorter LOS which is consistent with findings from prior research examining psychiatric hospitalizations (58, 68, 69). Specifically, hospital LOS in the pre-COVID and during-COVID periods was 69% and 55% shorter, respectively, for patients with substance-related disorders compared to those without substance-related disorders. The reduced hospital LOS may be attributed to various factors, including altered discharge planning processes, or modifications/reductions in the overall healthcare system capacity (e.g., when demand is not met with additional capacity, this can translate into shorter hospital stays) (70); however, it is important to consider that the observed decrease in LOS may also reflect changes in case mix or individual care needs during the pandemic. While substance use has been linked to shorter hospital LOS, further evaluation and examination of the impact of shorter LOS for people with a substance use disorder are warranted in the context of documented unmet treatment needs (71). These findings contribute to the existing body of literature on psychiatric disorders and LOS, emphasizing the importance of providing evidence-based care that addresses the specific needs of individuals with psychiatric and substance use disorders.

While our study has several strengths including a large sample and use of administrative data, there are some limitations to consider. Firstly, our results may not be generalizable to other settings or populations, as they are based on data from a single site. Given the location of the study site, our sample may not be representative of the broader population, leading to potential selection biases and limited external validity. Secondly, administrative data primarily captures information that is routinely collected for billing purposes, which may not capture all relevant clinical variables or detailed patient characteristics. Data describing race/ethnicity were not reliably collected as part of routine care and therefore excluded from our analysis. This can result in incomplete or limited data on potential confounding variables, making it challenging to fully account for all factors that could influence the outcome of interest. Thirdly, the use of administrative data is subject to potential data quality issues, including inaccuracies, missing data, and coding errors. These issues can introduce measurement biases and affect the validity of the study findings. Fourthly, we could not examine the circumstance surrounding admission such as whether pandemic related restrictions contributed and our data does not capture all relevant parameters contributing to LOS such as community services and social or family supports which may have impacted LOS. Researchers should consider these limitations when interpreting both the magnitude and direction of the effect size, and generalize the findings cautiously to other populations or settings. Future research would benefit from additional analysis to account for social context, changes during the early pandemic period and the inclusion of multiple study sites.

## Conclusions

We observed a significant difference in length of stay for mental and substance use disorders during the COVID-19 period. These findings underscore the need for further evaluation of the healthcare system to effectively plan and adapt to evolving utilization patterns during pandemics and public health emergencies, particularly for psychiatric populations. Our results provide knowledge about determinants of LOS which could lead to improvements in the quality of psychiatric hospital care for people with mental and substance use disorders. Future research is needed to understand the long-term implications of pandemic-related changes in mental illness and related healthcare utilization.

## Data Availability

The data analyzed in this study is subject to the following licenses/restrictions: The datasets analyzed during the current study are not publicly available due to privacy reasons. Requests to access these datasets should be directed to arussolillo1@providencehealth.bc.ca.
