# Supplementary material for: Adult psychiatric inpatient admissions and length of stay before and during the COVID-19 pandemic in a large urban hospital setting in Vancouver, British Columbia
Source: Front Health Serv. 2024 May 14;4:1365785. doi: 10.3389/frhs.2024.1365785 (PMC11130439; doi:10.3389/frhs.2024.1365785)
Supplement: Supplementary file 1 [file Datasheet1.docx]

**Supplementary Material**

Russolillo A, Carter M, Guan M, Singh P, Kealy D and Raudzus J (2024) Adult psychiatric inpatient admissions and length of stay before and during the COVID-19 pandemic in a large

urban hospital setting in Vancouver, British Columbia. Front. Health Serv. 4:1365785.

doi: 10.3389/frhs.2024.1365785

**Figure S1** Distribution of hospital LOS (days) for patients with and without schizophrenia, delusional and non-organic psychotic disorders at the pre-COVID (2019; n=472) and during-COVID period (2020; n=467) from an urban hospital in Vancouver, BC

**Figure S2** Distribution of hospital LOS (days) for patients with and without substance-related disorders at the pre-COVID (2019; n=472) and during-COVID period (2020; n=467) from an urban hospital in Vancouver, BC

**Figure S3** Distribution of hospital LOS for patients with and without mood-affective disorders at the pre-COVID (2019; n=472) and during-COVID period (2020; n=467) from an urban hospital in Vancouver, BC

**Figure S4** Distribution of hospital LOS for patients with and without anxiety disorders at the pre-COVID (2019; n=472) and during-COVID period (2020; n=467) from an urban hospital in Vancouver, BC

**Figure S5** Distribution of hospital LOS for patients with and without adult personality and behaviour disorders at the pre-COVID (2019; n=472) and during-COVID period (2020; n=467) from an urban hospital in Vancouver, BC

**Table S1** Distribution of hospital LOS (days) by pre-COVID period (2019) and during-COVID period (2020) and by diagnosis types for patients from an urban hospital in Vancouver, BC

This supplementary material has been provided by the authors to giver readers’ additional information about their work. Additional details related to site specific COVID-19 policies are available upon request.

**Figure S1** Distribution of hospital LOS (days) for patients with and without schizophrenia, delusional and non-organic psychotic disorders at the pre-COVID (2019; n=472) and during-COVID period (2020; n=467) from an urban hospital in Vancouver, BC

**
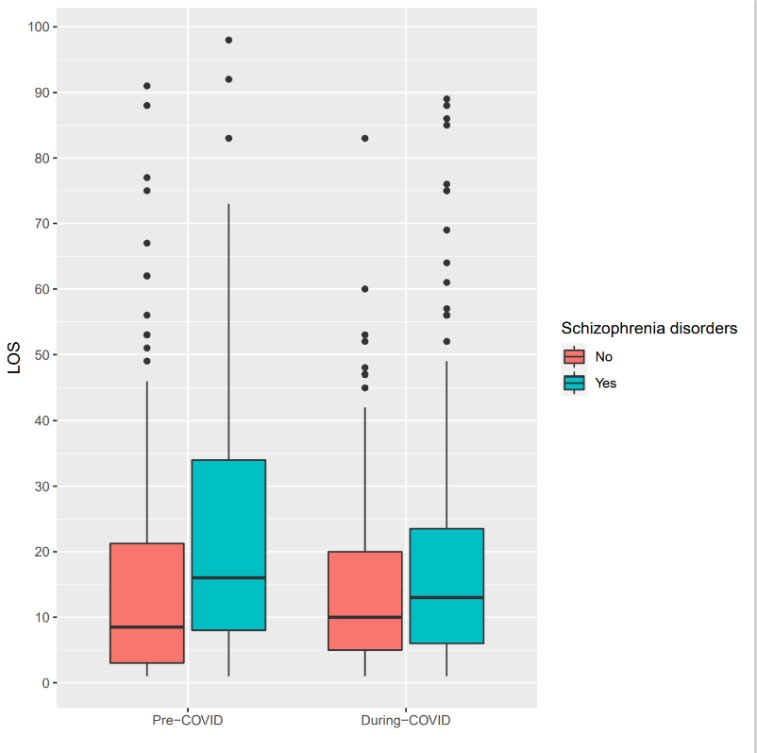
**

**Figure S2** Distribution of hospital LOS (days) for patients with and without substance-related disorders at the pre-COVID (2019; n=472) and during-COVID period (2020; n=467) from an urban hospital in Vancouver, BC

**
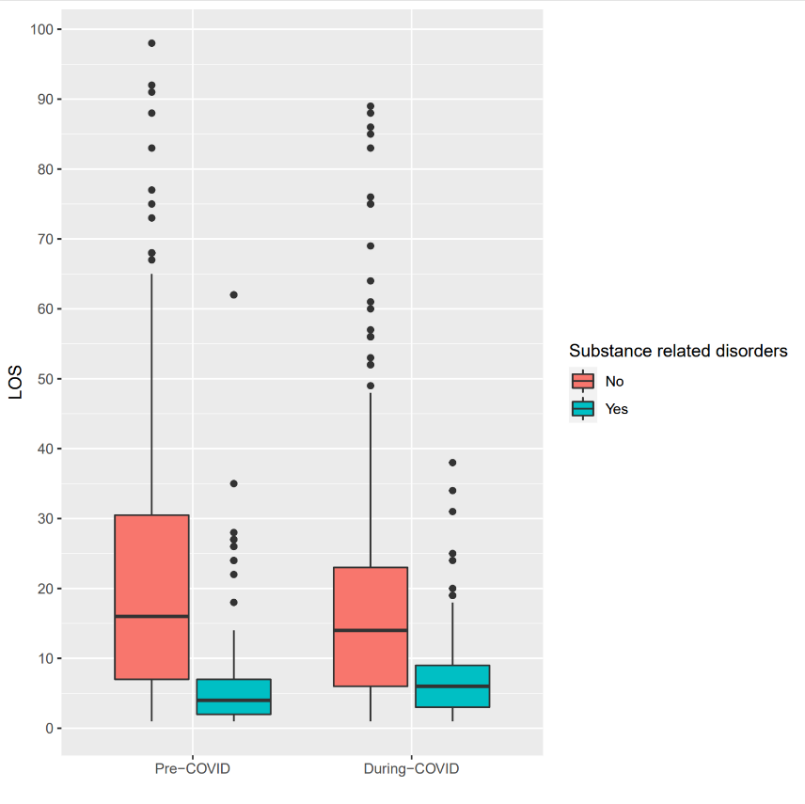
**

**Figure S3** Distribution of hospital LOS for patients with and without mood-affective disorders at the pre-COVID (2019; n=472) and during-COVID period (2020; n=467) from an urban hospital in Vancouver, BC

**
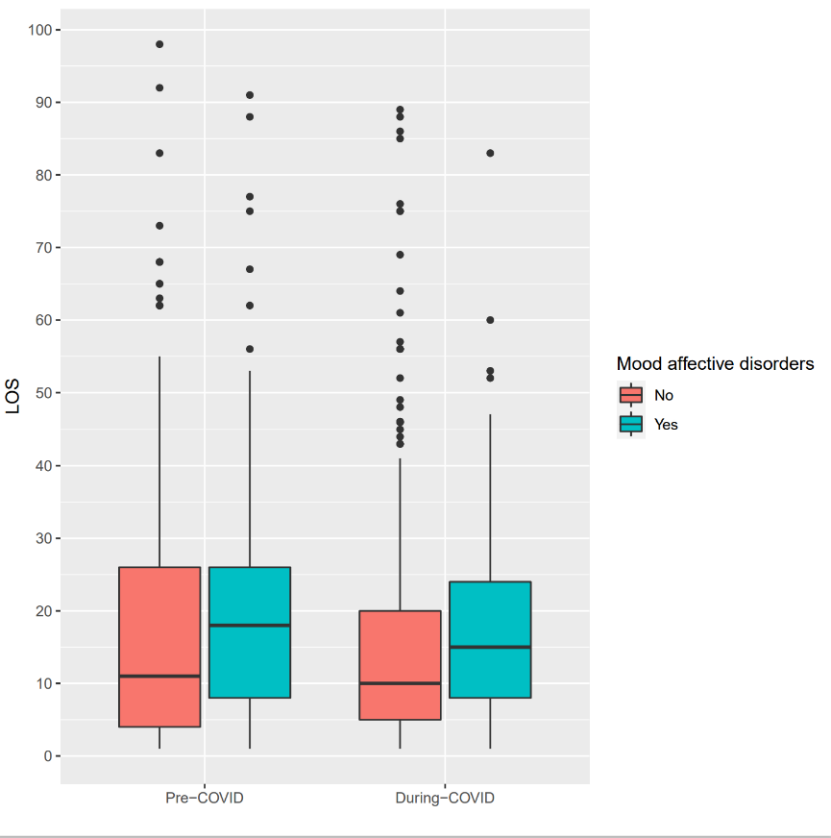
**

**Figure S4** Distribution of hospital LOS for patients with and without anxiety disorders at the pre-COVID (2019; n=472) and during-COVID period (2020; n=467) from an urban hospital in Vancouver, BC

**
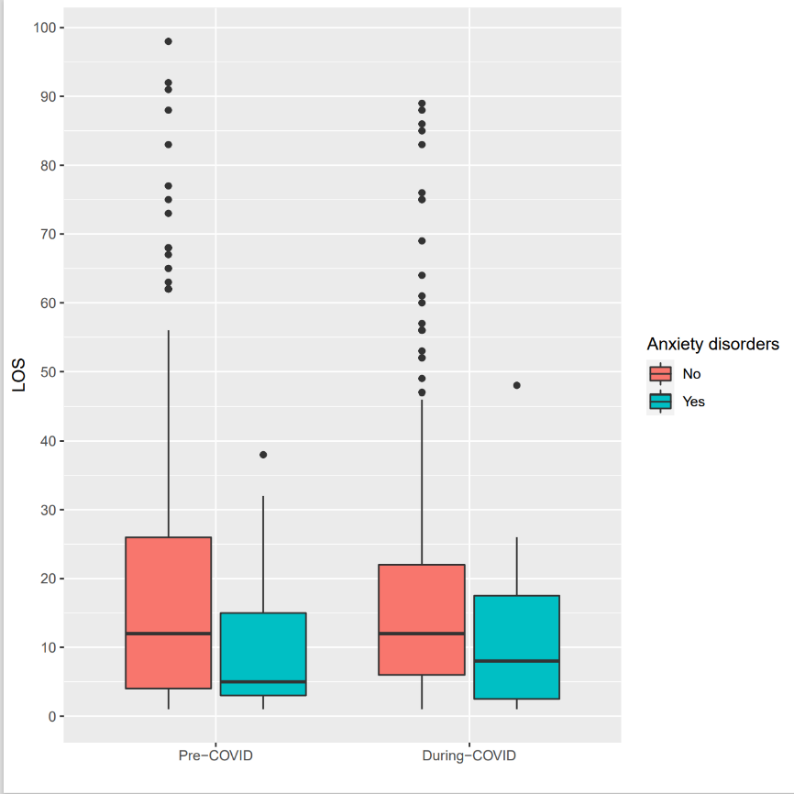
**

**Figure S5** Distribution of hospital LOS for patients with and without adult personality and behaviour disorders at the pre-COVID (2019; n=472) and during-COVID period (2020; n=467) from an urban hospital in Vancouver, BC

**
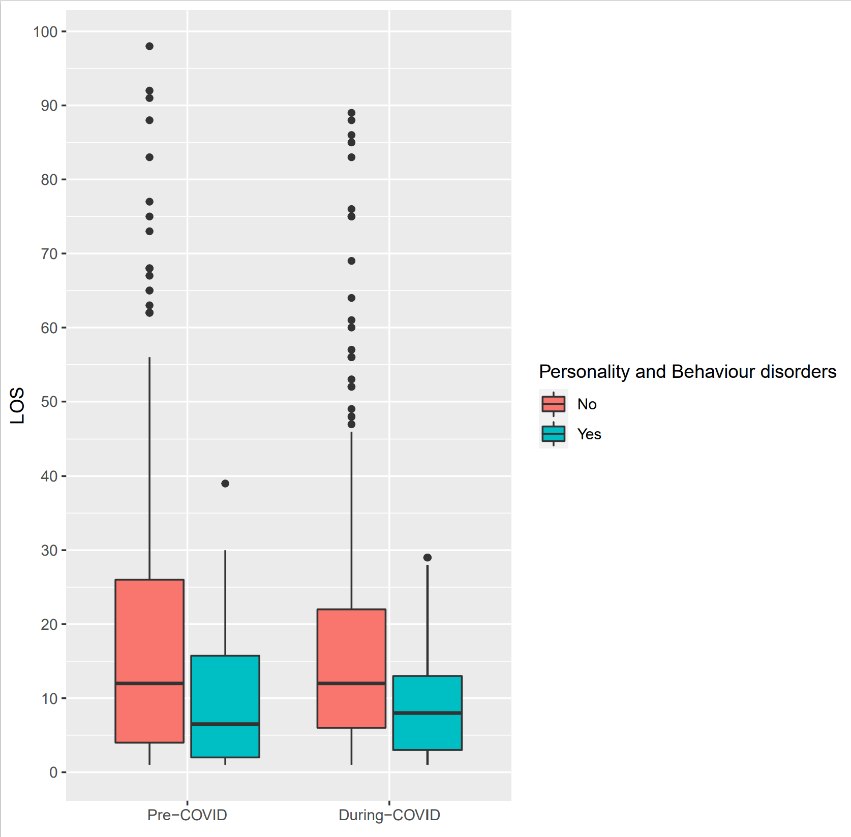
**

**Table S1** Distribution of hospital LOS (days) by pre-COVID period (2019) and during-COVID period (2020) and by diagnosis types for patients from an urban hospital in Vancouver, BC.

| Period | Diagnosis type | LOS (days) | | | | | | | | P-value^[[1]](#footnote-1)^ |
| --- | --- | --- | --- | --- | --- | --- | --- | --- | --- | --- |
|  |  | N | Min | Q1 | Mean | Median | SD | Q3 | Max |  |
|  | Schizophrenia, delusional and non-organic psychotic disorders |  |  |  |  |  |  |  |  | 0.390 |
| Pre-COVID | Yes | 193 | 1 | 8 | 24.9 | 17.0 | 24.3 | 35 | 171 |  |
|  | No | 279 | 1 | 3 | 17.6 | 10.0 | 24.3 | 23 | 190 |  |
| During-COVID | Yes | 232 | 1 | 6 | 19.0 | 13.5 | 19.6 | 24 | 153 |  |
|  | No | 235 | 1 | 5 | 14.1 | 10.0 | 12.9 | 20 | 83 |  |
|  | Substance-related disorders |  |  |  |  |  |  |  |  | 0.057 |
| Pre-COVID | Yes | 102 | 1 | 2 | 7.6 | 4.0 | 10.5 | 7 | 62 |  |
|  | No | 370 | 1 | 8 | 24.2 | 16.0 | 26.0 | 32 | 190 |  |
| During-COVID | Yes | 72 | 1 | 3 | 8.1 | 6.0 | 7.8 | 9 | 38 |  |
|  | No | 395 | 1 | 6 | 18.1 | 14.0 | 17.4 | 23 | 153 |  |
|  | Mood-affective disorders |  |  |  |  |  |  |  |  | 0.254 |
| Pre-COVID | Yes | 128 | 1 | 8 | 24.4 | 19.0 | 26.9 | 27.5 | 190 |  |
|  | No | 344 | 1 | 4 | 19.2 | 11.0 | 23.5 | 27 | 171 |  |
| During-COVID | Yes | 129 | 1 | 8 | 17.7 | 15.0 | 13.6 | 24 | 83 |  |
|  | No | 338 | 1 | 5 | 16.1 | 10.5 | 17.7 | 20 | 153 |  |
|  | Anxiety disorders |  |  |  |  |  |  |  |  | 0.478 |
| Pre-COVID | Yes | 17 | 1 | 3 | 11.5 | 5.0 | 11.3 | 15 | 38 |  |
|  | No | 455 | 1 | 5 | 21.0 | 13.0 | 24.8 | 28 | 190 |  |
| During-COVID | Yes | 7 | 1 | 2 | 13.9 | 8.0 | 17.3 | 26 | 48 |  |
|  | No | 460 | 1 | 6 | 16.6 | 12.0 | 16.7 | 22 | 153 |  |
|  | Adult personality and behavior disorders |  |  |  |  |  |  |  |  | 0.652 |
| Pre-COVID | Yes | 17 | 1 | 2 | 17.4 | 8.0 | 28.0 | 18 | 117 |  |
|  | No | 455 | 1 | 5 | 20.7 | 13.0 | 24.4 | 27 | 190 |  |
| During-COVID | Yes | 15 | 1 | 2 | 9.9 | 8.0 | 8.9 | 14 | 29 |  |
|  | No | 452 | 1 | 6 | 16.8 | 12.0 | 16.9 | 22 | 153 |  |

Min: minimum; SD: standard deviation; Max: maximum

1. A negative binomial model was used to test the interaction between diagnosis type yes vs no and pre-COVID vs during COVID on hospital LOS. A p-value <0.1 for the interaction was considered statistically significant. [↑](#footnote-ref-1)
